# Molecular and Cellular Mechanisms Responsible for Beneficial Effects of Mesenchymal Stem Cell-Derived Product “Exo-d-MAPPS” in Attenuation of Chronic Airway Inflammation

**DOI:** 10.1155/2020/3153891

**Published:** 2020-03-20

**Authors:** Carl Randall Harrell, Dragica Miloradovic, Ruxana Sadikot, Crissy Fellabaum, Bojana Simovic Markovic, Dragana Miloradovic, Aleksandar Acovic, Valentin Djonov, Nebojsa Arsenijevic, Vladislav Volarevic

**Affiliations:** ^1^Regenerative Processing Plant, LLC, 34176 US Highway 19 N, Palm Harbor, Florida, USA; ^2^Center for Molecular Medicine and Stem Cell Research, Department for Microbiology and Immunology, Faculty of Medical Sciences, University of Kragujevac, 69 Svetozar Markovic Street, Kragujevac, Serbia; ^3^Emory University School of Medicine, 648 Pierce Dr. NE, Atlanta, GA, USA; ^4^Atlanta VA Medical Center, 1670 Clairmont Rd., Decatur/Atlanta, GA, USA; ^5^Institute of Anatomy, University of Bern, 2 Baltzerstrasse, Switzerland

## Abstract

Mesenchymal stem cells (MSCs), due to their potential for differentiation into alveolar epithelial cells and their immunosuppressive characteristics, are considered a new therapeutic agent in cell-based therapy of inflammatory lung disorders, including chronic obstructive pulmonary disease (COPD). Since most of the MSC-mediated beneficent effects were the consequence of their paracrine action, herewith, we investigated the effects of a newly designed MSC-derived product “Exosome-derived Multiple Allogeneic Protein Paracrine Signaling (Exo-d-MAPPS)” in the attenuation of chronic airway inflammation by using an animal model of COPD (induced by chronic exposure to cigarette smoke (CS)) and clinical data obtained from Exo-d-MAPPS-treated COPD patients. Exo-d-MAPPS contains a high concentration of immunomodulatory factors which are capable of attenuating chronic airway inflammation, including soluble TNF receptors I and II, IL-1 receptor antagonist, and soluble receptor for advanced glycation end products. Accordingly, Exo-d-MAPPS significantly improved respiratory function, downregulated serum levels of inflammatory cytokines (TNF-*α*, IL-1*β*, IL-12, and IFN-*γ*), increased serum concentration of immunosuppressive IL-10, and attenuated chronic airway inflammation in CS-exposed mice. The cellular makeup of the lungs revealed that Exo-d-MAPPS treatment attenuated the production of inflammatory cytokines in lung-infiltrated macrophages, neutrophils, and natural killer and natural killer T cells and alleviated the antigen-presenting properties of lung-infiltrated macrophages and dendritic cells (DCs). Additionally, Exo-d-MAPPS promoted the expansion of immunosuppressive IL-10-producing alternatively activated macrophages, regulatory DCs, and CD4+FoxP3+T regulatory cells in inflamed lungs which resulted in the attenuation of chronic airway inflammation. In a similar manner, as it was observed in an animal model, Exo-d-MAPPS treatment significantly improved the pulmonary status and quality of life of COPD patients. Importantly, Exo-d-MAPPS was well tolerated since none of the 30 COPD patients reported any adverse effects after Exo-d-MAPPS administration. In summing up, we believe that Exo-d-MAPPS could be considered a potentially new therapeutic agent in the treatment of chronic inflammatory lung diseases whose efficacy should be further explored in large clinical trials.

## 1. Introduction

COPD is a common, progressive, inflammatory disease characterized by persistent and not fully reversible airflow limitation associated with an abnormal and deregulated chronic inflammatory response to noxious particles or gases [1–3]. Hallmarks of early stages of COPD include remodeling of small airways induced by persistently activated innate immune cells (alveolar macrophages, neutrophils, natural killer (NK), and DCs), while severe stages of COPD are characterized by the development of lung lymphoid follicles due to the enhanced activation of CD8+ cytotoxic T lymphocytes (CTLs) and CD4+ T helper cells and their crosstalk with B cells [4–8]. Continuous activation of resident and lung-infiltrated immune cells results in structural and functional changes in the inflamed lungs, including the narrowing of small airways, mucus hyperproduction, cilia dysfunction, and destruction of the lung parenchyma [9]. Accordingly, COPD is manifested by persistent and progressive breathlessness (usually worse during physical activities), chronic cough, regular production of sputum (in three or more months during two consecutive years), wheeze, and chest tightness (as signs of severe COPD) [9].

In a similar manner, as it is observed in human pathology, cigarette smoke (CS) exposure results in the development of COPD in mice [10]. Therefore, mouse models are usually used to elucidate molecular and cellular mechanisms involved in the development and progression of human COPD [10]. Importantly, there are strain-dependent differences in susceptibility to COPD-related injury between BALB/c and C57BL/6 mice. BALB/c mice were more sensitive than C57BL/6 mice to COPD-related emphysema, one of the major components of CS-induced COPD [11]. Significantly higher mortality, greater body weight loss, greater decline in lung function, and a greater loss of alveolar tissue were noticed in BALB/c mice compared to C57BL/6 mice [11]. Importantly, immune cells that play a crucially important pathogenic role in the development of cigarette smoke-induced COPD (TNF-*α*-producing, classically activated (M1) macrophages and neutrophils, as well as IL-17-producing lymphocytes) were found in significantly higher numbers in the injured lungs of BALB/c mice compared to C57BL/6 mice, indicating the advantage of using BALB/c mice in COPD-related animal studies [10–12].

Available medicaments (anti-inflammatory drugs, *β*2-agonists, and anticholinergics) efficiently reduce airflow limitation and improve the quality of life of COPD patients, but they are not able to prevent disease progression and mortality [13]. Since COPD is projected to become the third leading cause of disease mortality worldwide by 2020 [1, 2], new therapeutic approaches are urgently needed in order to prevent progression and exacerbation of COPD.

MSCs are self-renewable, multipotent cells which are able to differentiate into alveolar epithelial cells and are able to modulate the proliferation, activation, and effector function of all immune cells that play an important role in the development, progression, and exacerbation of COPD [14]. Accordingly, several experimental studies demonstrated the beneficial effects of MSCs in the treatment of COPD [15–18]. Intravenously, intratracheally, and intrabronchially injected MSCs managed to significantly attenuate emphysematous changes and notably improve pulmonary function in animal models of COPD [19]. The beneficial effects of MSCs were mainly attributed to the activity of MSC-derived products (conditioned medium (MSC-CM) and exosomes (MSC-Exos)): nanosized extracellular vesicles that, in a paracrine and endocrine manner, delivered proteins, lipids, DNA fragments, and mRNA to the immune, endothelial, and alveolar epithelial cells modulating their function [20]. Several lines of evidence suggested that MSCs derived from placental tissues (PL-MSCs) had superior biological properties compared to MSCs derived from adult tissues [21–24]. In line with these findings, we recently developed the “Exosome-derived Multiple Allogeneic Protein Paracrine Signaling (Exo-d-MAPPS),” a PL-MSC-derived product whose activity is based on exosomes, growth factors, and immunomodulatory cytokines capable of attenuating inflammation and promoting the regeneration of injured tissues [25]. Herewith, we described the molecular and cellular mechanisms which were responsible for the Exo-d-MAPPS-based attenuation of airway inflammation in BALB/c mice and in patients suffering from COPD.

## 2. Material and Methods

### 2.1. Exo-d-MAPPS Sample Acquisition

Sterile Exo-d-MAPPS is an engineered biological product obtained from PL-MSCs previously collected from healthy human donors. PL-MSC samples were obtained with patient consent as well as institutional ethical approval and kept at 4°C until processed. All donors prior to or at the time of collection were tested by laboratories certified under the Clinical Laboratory Improvement Amendments (CLIA) and were found negative using United States (US) Food and Drug Administration (FDA) licensed tests for the detection of at minimum, hepatitis B virus, hepatitis C virus, human immunodeficiency virus types 1/2, and *Treponema pallidum*.

The Exo-d-MAPPS samples used in this study were manufactured under specific conditions in order to be applicable for bioavailability testing and for different therapeutic uses. The Exo-d-MAPPS sample was engineered as a sterile product and manufactured under current Good Manufacturing Practices (cGMP) regulated and reviewed by the FDA. The sterile Exo-d-MAPPS sample incorporates the Regenerative Processing Plant's (RPP) proprietary patented sterilization process to provide a safe sterile product [25]. Briefly, PL-MSCs were grown in complete MSC Dulbecco's Modified Eagle's Medium (DMEM). Low passage (<5) PL-MSCs were grown to 60%–80% confluence in multiflasks before isolation. Fresh PL-MSC media were layered and collected after 48 to 72 h (conditioned medium). Exos were isolated by the ultracentrifugation protocol (100,000g at 4°C for 70 min). The isolation of Exos was performed by positive selection using the *μ*MACS™ Separator (Miltenyi Biotec, Bergisch Gladbach, Germany; Cat. No. 130-042-602) and the Exosome Isolation Kit Pan, human (Miltenyi Biotec, Bergisch Gladbach, Germany; Cat. No. 130-110-912) which contained a cocktail of MicroBeads conjugated to the tetraspanin proteins CD9, CD63, and CD81. Briefly, Exos were magnetically labeled and loaded onto a *μ* column, which was placed in the magnetic field of a *μ*MACS™ Separator. The magnetically labeled Exos were retained within the column, while the unlabeled vesicles and cell components run through the column. After removing the column from the magnetic field, the intact Exos were collected by elution. Exos were stored at −70°C until use [25].

### 2.2. Animals

For animal studies, eight- to ten-week-old male BALB/c mice were used. Mice were maintained in animal facilities of the Faculty of Medical Sciences, University of Kragujevac, Serbia. All animals received humane care, and all experiments were approved by and conducted in accordance with the Guidelines of the Animal Ethics Committee of the Faculty of Medical Sciences of the University of Kragujevac. Mice were housed in a temperature-controlled environment with a 12-hour light-dark cycle and were administered with standard laboratory chow and water ad libitum.

### 2.3. Animal Study Protocol

Animals were randomly divided into control and experimental groups (*n* = 8 mice per group). Mice from the experimental group underwent whole-body exposure to CS of 5 cigarettes in a CS chamber with 30-minute smoke-free intervals, every day for four weeks [26, 27]. The smoke exposure experimental box, adapted for a group of 8 mice, consisted of a box body and a cover. CS was drawn through an exposure chamber by negative pressure using an extraction pump. Between draws of CS, room air was continuously drawn through the chamber. The smoke-to-air ratio was 1 : 12 to protect mice from acute smoke toxicity and death.

After four weeks of CS treatment, mice were randomly divided into two groups and received either vehicle or Exo-d-MAPPS (0.1 mL/intraperitoneally/5 days per week for three weeks).

Mice from the control groups were exposed to air only and received either vehicle or Exo-d-MAPPS.

### 2.4. Histopathological Analysis

All mice were sacrificed 8 weeks after initial CS exposure, and the lungs were isolated for histopathological analysis. The isolated lungs were fixed in 10% formalin, embedded in paraffin, and consecutive 4 *μ*m tissue sections were mounted on slides. Sections were stained with hematoxylin and eosin (H&E) and examined under a low-power (100x) light microscope-equipped digital camera (Zeiss Axioskop 40, Jena, Germany) [28].

### 2.5. Blood Gas Analysis

In order to explore whether Exo-d-MAPPS treatment managed to improve extracellular acid-base status and gas exchange in CS-exposed mice, blood gas parameters (partial pressure of oxygen in arterial blood (PaO_2_), partial pressure of carbon dioxide (PaCO_2_) in arterial blood, oxygen saturation (SaO_2_), and pH) were analyzed. For this purpose, arterial blood samples were obtained from control and experimental animals and analyzed within a few minutes using a test cartridge blood analysis system (Premier GEM 3500, Instrumentation Laboratory, Bedford, Massachusetts, USA) [29].

### 2.6. Isolation of Lung-Infiltrated Immune Cells

The lungs, obtained from control and CS-exposed mice, were washed with sterile phosphate-buffered saline (PBS) and placed in Petri dishes with DMEM supplemented with 10% FBS. The dissected lung tissues were incubated in a medium that contained collagenase type IV (0.5 mg/mL) and type IV bovine pancreatic DNAse (Roche Diagnostics; 1 mg/mL) at 37°C for 45 min. The cells were filtered through a 100 *μ*m nylon cell strainer into a clean 50 mL conical tube. Then, cells were pelleted by centrifuging for 10 min at 300 × g at 10°C. Red blood cells were depleted with a lysis buffer (0.144 M NH4Cl, 0.0169 M TRIS base, pH 7.4) at 37°C in a 5% CO_2_ atmosphere for 5 min [28].

### 2.7. Flow Cytometry Analysis and Intracellular Staining of Lung-Infiltrated Immune Cells

Lung-infiltrated immune cells were screened for various cell surface and intracellular markers by flow cytometry. Since a combination of mechanical and enzymatic dissociations of lung tissue may result in cell damage and death, the MACS® Dead Cell Removal Kit (Miltenyi Biotec, Bergisch Gladbach, Germany; Cat. No. 130-090-101) was used for magnetic cell separation of viable cells. Briefly, a single-cell suspension of lung-infiltrated cells was resuspended in 100 *μ*L of the Dead Cell Removal MicroBeads (per 10^7^ of cells), mixed, and incubated for 15 min at room temperature. Cells were applied on MS columns within 1 × MACS Binding Buffer. Effluent that passed through the column contained live cells. To reduce nonspecific binding of antibodies, viable lung-infiltrated cells were incubated with an anti-Fc block (anti-mouse CD16/CD32). For that purpose, the cell suspension was incubated with 1 *μ*g of the BD Fc Block/10^6^ cells in 100 *μ*L of staining buffer (Dulbecco's PBS (DPBS) without Mg^2+^ or Ca^2+^, 1% heat-inactivated FCS, and 0.09% (*w*/*v*) sodium azide) for 15 minutes at 4°C. The cells were then washed and stained with fluorochrome-conjugated antibodies. Briefly, 1 × 10^6^ cells were incubated with anti-mouse CD45, F4/80, I-A, CD80, CD206, CD11c, NKp46, Gr-1, CD3, CD4, CD8, CXCR3, monoclonal antibodies conjugated with fluorescein isothiocyanate (FITC), phycoerythrin (PE), peridinin chlorophyll protein (PerCP), or allophycocyanin (APC) (all from BD Biosciences, San Jose, CA, USA) in a staining buffer for 30 min in the dark at 4°C. Cells were washed twice in a staining buffer and pelleted by centrifugation. For intracellular cytokine staining, cells were stimulated with 50 ng/mL phorbol 12-myristate 13-acetate (PMA) and 500 ng/mL ionomycin for 5 h and GolgiStop (BD Biosciences, San Jose, CA, USA; Cat. No. 554715) was added. Cells were then incubated in a BD fixation/permeabilization solution (BD Cytofix/Cytoperm™ Fixation/Permeabilization Kit; Cat. No. 554714) for 20 min at 4°C. Afterwards, cells were washed two times in 1 × BD Perm/Wash™ buffer (BD Cytofix/Cytoperm™ Fixation/Permeabilization Kit; Cat. No. 554714) and pelleted. Fixed/permeabilized cells were concomitantly resuspended in 50 *μ*L of BD Perm/Wash™ buffer containing a predetermined optimal concentration of fluorochrome-conjugated antibodies specific for FoxP3, TNF-*α*, IL-12, IL-10, IL-1*β*, IFN-*γ*, and IL-17 by using appropriate anti-mouse monoclonal antibodies conjugated with FITC, PE, PerCP, and APC (BD Biosciences, San Jose, CA, USA). Cells were incubated with fluorochrome-conjugated antibodies at 4°C for 30 minutes in the dark. Afterwards, cells were washed 2 times with 1 × BD Perm/Wash™ buffer and resuspended in a staining buffer prior to flow cytometric analysis. In experiments in which the phenotype and function of T cells were analyzed, CD3+ T lymphocytes were isolated from the population of viable lung-infiltrated cells by magnetic separation. For that purpose, the MACS Separator, the MACS Columns, and the CD3*ε* MicroBead Kit, mouse (Miltenyi Biotec, Bergisch Gladbach, Germany; Cat. No. 130-094-973) were used. Afterwards, CD3+ T cells were stained with fluorochrome-conjugated anti-mouse antibodies specific for CD4, CD8, CXCR3, FoxP3, TNF-*α*, IL-10, IFN-*γ*, and IL-17, following the procedure that was described above. Flow cytometric analysis was conducted on a BD Biosciences' FACSCalibur and analyzed by using the Flowing Software analysis program [28].

### 2.8. Determination of Cytokines in Serum Samples of Control and Experimental Animals

The commercial ELISA sets (R&D Systems, Minneapolis, MN, USA) were used to determine the concentration of TNF-*α*, IL-12, IL-10, IL-1*β*, and IFN-*γ* in serum samples of control and experimental animals [28].

### 2.9. Patients

Thirty COPD patients were recruited with the aim to receive an Exo-d-MAPPS inhalation solution. Patients enrolled in this study were men (*n* = 20) or postmenopausal women (*n* = 10) aged between 50 and 75 years, having a postbronchodilator forced expiratory volume in 1 s (FEV1) ≥ 30% and <80% predicted, a postbronchodilator FEV1/forced vital capacity (FVC) < 70%, a smoking history of ≥10 pack-year, and lung hyperinflation defined as a functional residual capacity (FRC) greater than 120%. Subjects with past or current history of abnormal vital signs, abnormal laboratory findings, clinically relevant ECG abnormalities, or cardiovascular conditions prior to screening were excluded from the study. All subjects provided written informed consent prior to study participation.

### 2.10. Clinical Study Protocol

Patients received Exo-d-MAPPS inhalation solution (0.5 mL/once per week for three weeks) containing a high concentration of immunosuppressive factors (soluble TNF receptors I and II (sTNFRI and sTNFRII), IL-1 receptor antagonist (IL-1Ra), and soluble receptor for advanced glycation end products (sRAGE)) [25, 30]. Pulmonary function tests and clinical findings were recorded before and 1 month after Exo-d-MAPPS treatment. Spirometry was performed according to recommendations from the American Thoracic Society guidelines [31, 32]. Forced expiratory volume in 1 second (FEV1) and peak expiratory flow (PEF) rate were recorded. Chest computed tomography (CT), standard clinical COPD questionnaire (CCQ) scoring, and 6-minute walking distance (6MWD) test as a submaximal test of aerobic capacity/endurance were used to determine the effects of Exo-d-MAPPS treatment, as previously described [33–35].

### 2.11. Statistical Analysis

The results obtained in the animal study were analyzed using the Student *t*-test. All data in animal studies were expressed as the mean ± standard error of the mean (SEM). The Wilcoxon signed-rank test was applied to demonstrate differences in pulmonary function of COPD patients before and after Exo-d-MAPPS treatment. Values of *P* < 0.05 were considered as statistically significant.

## 3. Results

### 3.1. Exo-d-MAPPS Attenuated Chronic Airway Inflammation in Mice

**Figure 1:**
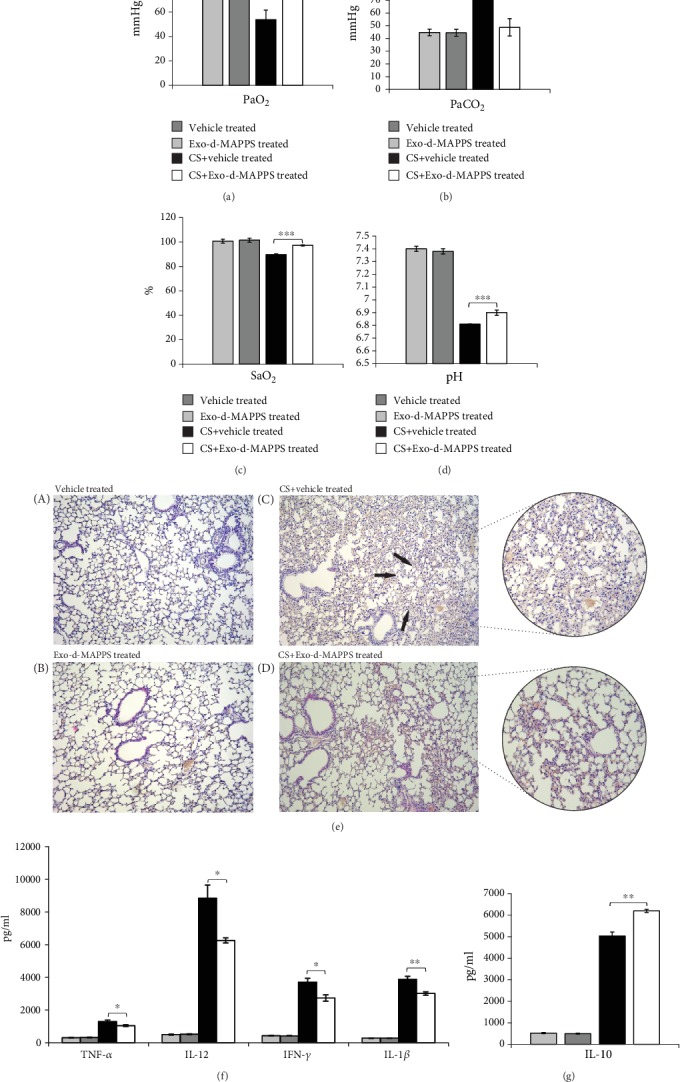
Exo-d-MAPPS attenuated CS-induced airway inflammation in mice. Exo-d-MAPPS significantly improved pulmonary function in CS-exposed mice, as evidenced by blood gas analysis: significantly elevated PaO_2_, SaO_2_, and pH and decreased PaCO_2_ (a–d). Representative H&E staining of the lungs (×100 magnification) obtained from the control ((e) A and (e) B) and experimental mice ((e) C and (e) D) showing partial alveolar wall destruction, widened alveolar septa and expanded alveolar space, and capillary dilation and congestion with massive infiltration of neutrophils, lymphocytes, and monocytes in the lungs of CS-exposed mice (e-C; black arrows) and alveolar and blood vessel structures with a lower number of lung-infiltrated leucocytes in the lungs of CS+Exo-d-MAPPS-treated animals (e-D). Significantly downregulated levels of proinflammatory cytokines (TNF-*α*, IL-12, IFN-*γ*, and IL-1*β*) (f) and increased concentration of anti-inflammatory IL-10 (g) in serum samples of CS+Exo-d-MAPPS-treated animals. Values are presented as mean ± SEM; *n* = 8 mice/group. ^∗^*P* < 0.05, ^∗∗^*P* < 0.01, and ^∗∗∗^*P* < 0.001.

The analysis of arterial blood gas parameters, including PaO_2_, PaCO_2_, pH, and SaO_2_ (Figures 1(a)–1(d)), indicated respiratory dysfunction in CS-exposed mice which was manifested by tiredness, fatigue, and reduced activity. Importantly, remarkably improved respiratory function, as evidenced by significantly elevated PaO_2_ (Figure 1(a), *P* < 0.0001), O_2_ saturation (Figure 1(c), *P* < 0.0001), and pH (Figure 1(d), *P* < 0.0001) and decreased PaCO_2_ (Figure 1(b), *P* < 0.0001) was observed in CS-treated mice that received Exo-d-MAPPS. Accordingly, depression-like behavior and loss of locomotor activity were not seen in CS+Exo-d-MAPPS-treated animals.

The alveolar wall was intact, and leucocyte accumulation was not seen in the lung parenchyma of control animals (Figures 1(e), A and 1(e), B). On the contrary, partial alveolar wall destruction, widened alveolar septa and expanded alveolar space, capillary dilation, and congestion with massive infiltration of neutrophils, lymphocytes, and monocytes were observed in the lungs of CS-exposed mice (Figure 1(e), C; black arrows). Importantly, preserved alveolar and blood vessel structures and a significantly lower number of lung-infiltrated leucocytes were noticed in the lungs of CS+Exo-d-MAPPS-treated animals (Figures 1(e) D) indicating that Exo-d-MAPPS managed to attenuate inflammation-related pathological changes in the lungs of CS-exposed mice.

In line with these findings, a significantly lower concentration of inflammatory cytokines that play an important pathogenic role in the development and progression of CS-induced airway inflammation (TNF-*α*, IL-1*β*, IL-12, and IFN-*γ*) was observed in serum samples of Exo-d-MAPPS-treated CS-exposed mice compared to CS+vehicle-treated animals (Figure 1(f); *P* < 0.05 for TNF-*α*, IL-12, and IFN-*γ*; *P* < 0.01 for IL-1*β*). Additionally, Exo-d-MAPPS treatment resulted in the elevation of anti-inflammatory and immunosuppressive IL-10 (Figure 1(g), *P* < 0.01) which is involved in lung repair and regeneration [4].

### 3.2. Exo-d-MAPPS Significantly Attenuated Influx of Inflammatory Macrophages, Neutrophils, and NK and NKT Cells in Inflamed Lungs

**Figure 2:**
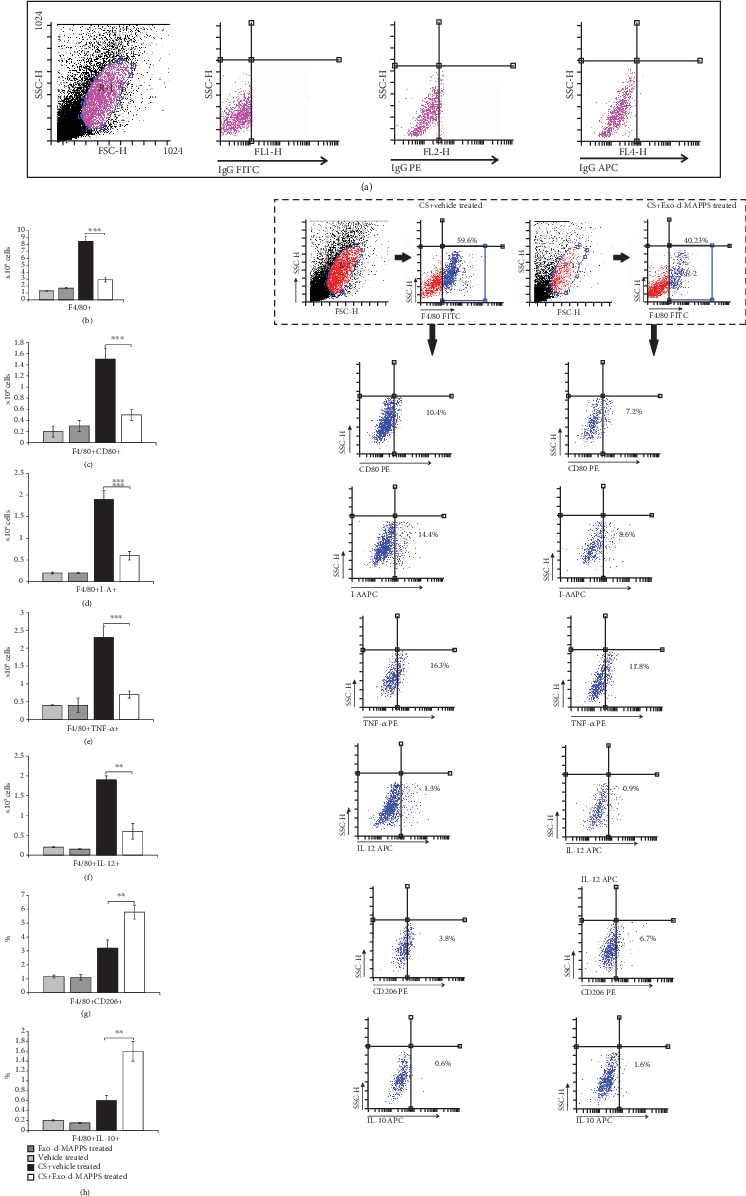
Exo-d-MAPPS treatment prevented the influx of inflammatory macrophages and induced the expansion of alternatively activated, IL-10-producing macrophages in the CS-injured lungs. Representative dot plots showing isotype controls (a). The total number of lung-infiltrated F4/80+ macrophages was significantly lower in CS+Exo-d-MAPPS-treated animals, as evidenced by representative flow cytometry plots (b). A significantly decreased number of CD80-expressing (c), I-A-expressing (d), and TNF-*α*- (e) and IL-12-producing (f) F4/80+macrophages were noticed in the lungs of CS+Exo-d-MAPPS-treated mice, as evidenced by representative flow cytometry plots. Representative dot plots showing a significantly increased number of alternatively activated, CD206-expressing, (g) and IL-10-producing F4/80+ macrophages (h) were observed in CS-exposed animals that received Exo-d-MAPPS. Values are presented as mean ± SEM; *n* = 8 mice/group. ^∗^*P* < 0.05, ^∗∗^*P* < 0.01, and ^∗∗∗^*P* < 0.001.

Macrophages have a crucially important role in the development and progression of CS-induced airway inflammation in mice, and their number corresponds to the extent of lung injury and inflammation [36]. Exo-d-MAPPS treatment managed to significantly reduce the total number of lung-infiltrated macrophages in CS-exposed mice (Figure 2(b), *P* < 0.001). Additionally, Exo-d-MAPPS remarkably attenuated antigen-presenting capacities of alveolar macrophages as evidenced by a significantly reduced number of CD80- and I-A-expressing F4/80+ cells in the lungs of CS+Exo-d-MAPPS-treated animals (Figures 2(c) and 2(d), *P* < 0.001). Intracellular staining revealed that Exo-d-MAPPS significantly attenuated the production of inflammatory TNF-*α* (Figure 2(e), *P* < 0.001) and IL-12 (Figure 2(f), *P* < 0.01) in lung-infiltrated macrophages. Furthermore, a significantly higher number of alternatively activated, IL-10-producing and CD206-expressing M2 macrophages were noticed in the lungs of Exo-d-MAPPS-treated CS-exposed mice (Figures 2(g) and 2(h), *P* < 0.01), indicating that Exo-d-MAPPS treatment suppressed inflammation and promoted the generation of an immunosuppressive phenotype in lung-infiltrated macrophages.

**Figure 3:**
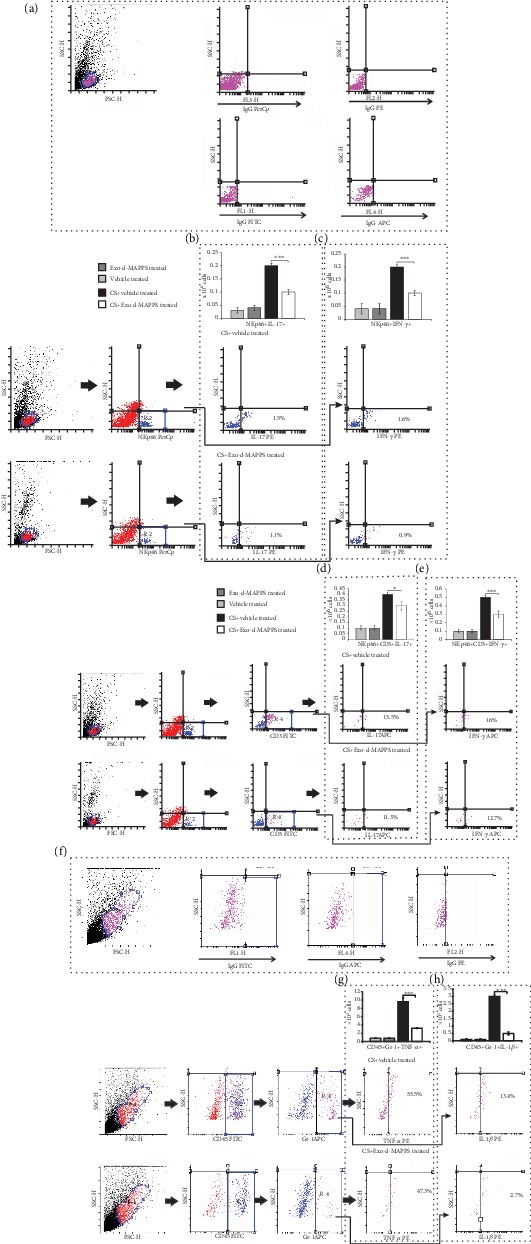
Exo-d-MAPPS attenuated the capacity of NK and NKT cells and neutrophils to produce inflammatory cytokines in CS-injured lungs. Representative dot plots showing isotype controls (a). The number of IL-17-producing (b, c) and IFN-*γ*-producing NK and NKT cells (d, e) was significantly lower in the lungs of CS+Exo-d-MAPPS-treated mice compared to CS+vehicle-treated animals, as evidenced by representative dot plots. Representative dot plots showing isotype controls gated on neutrophils (f).Exo-d-MAPPS significantly reduced the influx of TNF-*α* and IL-1*β*-producing CD45+Gr-1+ neutrophils in CS-injured lungs (g, h). Values are presented as mean ± SEM; *n* = 8 mice/group. ^∗^*P* < 0.05, ^∗∗^*P* < 0.01, and ^∗∗∗^*P* < 0.001.

Additionally, Exo-d-MAPPS attenuated the capacity of NK and NKT cells and neutrophils to produce inflammatory cytokines in CS-injured lungs. A significantly lower number of IL-17A-producing NK and NKT cells (Figures 3(b) and 3(c), *P* < 0.001 for NK and *P* < 0.05 for NKT cells), IFN-*γ*-secreting NK and NKT cells (Figures 3(d) and 3(e), *P* < 0.001), and TNF-*α* and IL-1*β*-producing neutrophils (Figures 3(g) and 3(h), *P* < 0.001) were observed in the lungs of Exo-d-MAPPS-treated CS-exposed mice.

### 3.3. Exo-d-MAPPS Significantly Alleviated Antigen-Presenting Properties of Lung-Infiltrated DCs That Resulted in Attenuated Activation of CD4+ and CD8+ T Lymphocytes

**Figure 4:**
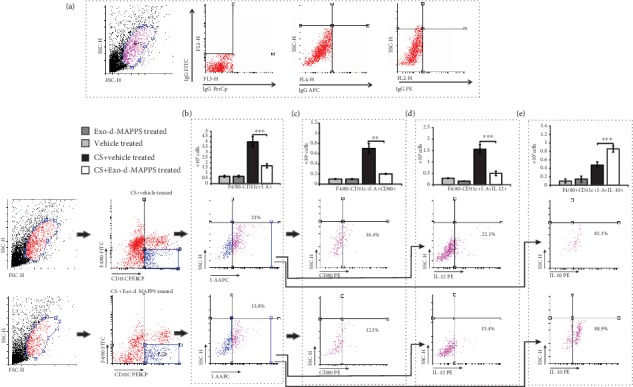
Exo-d-MAPPS reduces the influx of inflammatory DCs and promotes the expansion of regulatory DCs in CS-injured lungs. Representative dot plots showing isotype controls (a). The total number of F4/80-CD11c+I-A+ DCs was significantly lower in the lungs of CS+Exo-d-MAPPS-treated animals, as evidenced by representative flow cytometry plots (b). A significantly decreased number of CD80-expressing (c) and IL-12-producing (d) inflammatory F4/80-CD11c+I-A+ DCs were observed in the lungs of CS+Exo-d-MAPPS-treated mice, as evidenced by representative flow cytometry plots. Exo-d-MAPPS treatment significantly increased the presence of regulatory, IL-10-producing F4/80-CD11c+I-A+ DCs in the lungs of CS-exposed animals, as evidenced by representative flow cytometry plots (e). Values are presented as mean ± SEM; *n* = 8 mice/group. ^∗^*P* < 0.05, ^∗∗^*P* < 0.01, and ^∗∗∗^*P* < 0.001.

Airway DCs initiate and orchestrate T cell-driven inflammation in CS-injured lungs [34]. Lung DCs form a sentinel network around the airways, capture antigens that pass through the injured epithelium, and become activated. Activated DCs migrate to the draining lymph nodes to convey antigenic information to specialized T lymphocytes, inducing the generation of effector CD4+ T helper cells and CD8+CTLs [37, 38]. As it is shown in Figure 4, Exo-d-MAPPS affected the migratory and antigen-presenting properties of DCs. A significantly lower number of F4/80-CD11c+I-A+ DCs were observed in the CS-injured lungs of Exo-d-MAPPS-treated animals (Figure 4(b), *P* < 0.001). The total number of lung-infiltrated F4/80-CD11c+I-A+ DCs that express costimulatory molecule CD80 (Figure 4(c), *P* < 0.01) was significantly lower in CS-treated mice that received Exo-d-MAPPS. Furthermore, a decreased number of proinflammatory, IL-12-producing F4/80-CD11c+I-A+ DCs (Figure 4(d), *P* < 0.001) and an increased presence of immunosuppressive and tolerogenic, IL-10-producing F4/80-CD11c+I-A+ DCs (Figure 4(e), *P* < 0.001) were observed in the lungs of CS+Exo-d-MAPPS-treated animals, indicating that Exo-d-MAPPS attenuated the antigen-presenting and proinflammatory properties of airway DCs.

**Figure 5:**
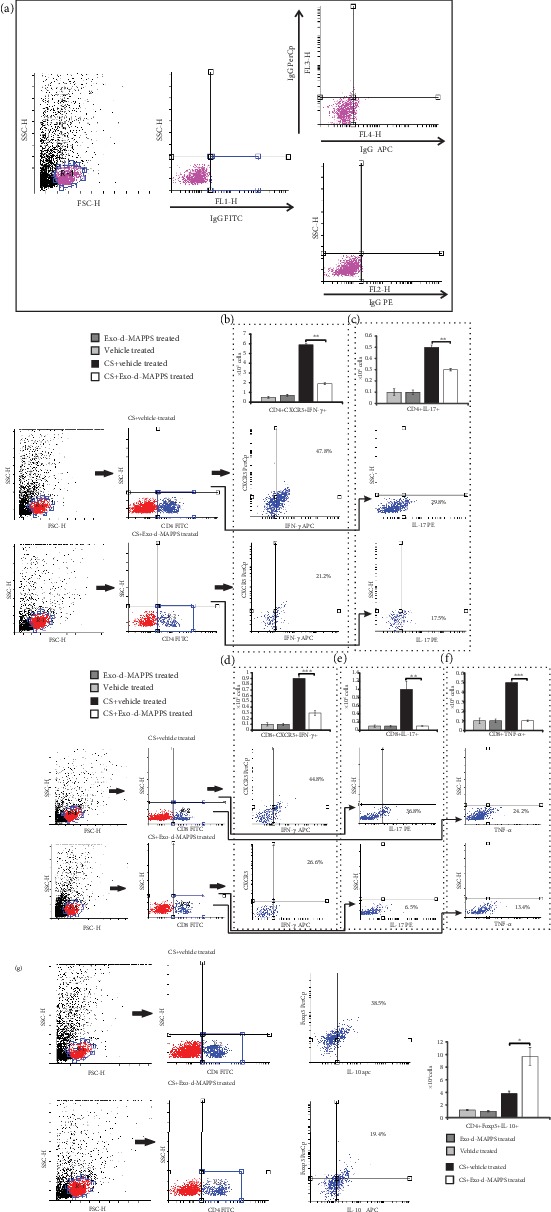
Exo-d-MAPPS significantly reduced the total number of inflammatory CD4+ and CD8+ T lymphocytes and increased the presence of immunosuppressive Tregs in inflamed lungs of CS-exposed animals. Representative dot plots showing isotype controls (a). A significantly lower number of CXCR3-expressing and IFN-*γ*-producing CD4+Th1 cells (b), IL-17-producing CD4+Th17 cells (c), CXCR3-expressing and IFN-*γ*-producing CD8+CTLs (d), and IL-17- (e) and TNF-*α*-producing CD8+CTLs (f) were noticed in the lungs of CS-treated mice that received Exo-d-MAPPS, as evidenced by representative dot plots. Exo-d-MAPPS treatment significantly increased the presence of FoxP3-expressing, IL-10-producing CD4+ Tregs in the lungs of CS-exposed animals, as evidenced by representative flow cytometry plots (g). Values are presented as mean ± SEM; *n* = 8 mice/group. ^∗^*P* < 0.05, ^∗∗^*P* < 0.01, and ^∗∗∗^*P* < 0.001.

Exo-d-MAPPS-induced modulation of DC function resulted in alleviated activation of inflammatory, IFN-*γ*- and IL-17-producing CD4+ and CD8+ T lymphocytes (Figures 5(b)–5(e)). A significantly lower number of CXCR3-expressing and IFN-*γ*-producing CD4+Th1 cells (Figure 5(b), *P* < 0.01) and IL-17-producing CD4+Th17 cells (Figure 5(c), *P* < 0.01) were observed in the lungs of Exo-d-MAPPS-treated CS-exposed mice. Similarly, Exo-d-MAPPS treatment attenuated the influx of CXCR-expressing, IFN-*γ*-producing (Figure 5(d), *P* < 0.001), and IL-17-producing CD8+CTLs (Figure 5(e), *P* < 0.01) and reduced the total number of alveolotoxic, TNF-*α*-producing CD8+CTLs (Figure 5(f), *P* < 0.001) in CS-injured lungs. Importantly, Exo-d-MAPPS significantly increased the total number of lung-infiltrated anti-inflammatory, IL-10-producing CD4+FoxP3+T regulatory cells (Tregs) (Figure 5(g), *P* < 0.05), enabling the generation of an immunosuppressive microenvironment in the inflamed lungs.

### 3.4. Exo-d-MAPPS Treatment Significantly Improved Pulmonary Status of COPD Patients

**Figure 6:**
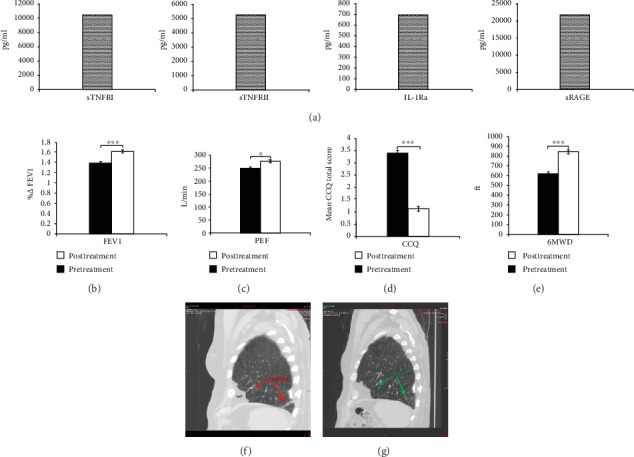
Exo-d-MAPPS treatment significantly improved the pulmonary status of COPD patients. Exo-d-MAPPS contains a high concentration of immunosuppressive factors (sTNFRI, sTNFRII, IL-1Ra, and sRAGE) (a). All of the 30 Exo-d-MAPPS-treated COPD patients showed marked improvement in pulmonary status, as evidenced by an increase in percentage change relative to initial value of FEV1 (%ΔFEV1), significantly higher PEF, decreased CCQ total score, and increased 6-minute walking distance (6MWD). Values are presented as mean ± SD. ^∗^*P* < 0.05, ^∗∗^*P* < 0.01, and ^∗∗∗^*P* < 0.001 (b e). The representative CT images showing less hyperexpanded lung, less flattened diaphragm, and reduced centrilobular and paraseptal emphysema in a COPD patient, one month after Exo-d-MAPPS treatment (f, g).

In order to investigate the relevance of the experimental findings for corresponding human pathology, we evaluated the efficacy of the Exo-d-MAPPS inhalation solution for the attenuation of airway inflammation in patients suffering from COPD (Figure 6). As it is shown in Figure 6(a), Exo-d-MAPPS contained a high concentration of soluble immunosuppressive mediators (sTNFRI, sTNFRII, IL-1Ra, and sRAGE). Clinical parameters (Figures 6(b)–6(e)) and CT findings (Figure 6(f)) indicated the beneficial effects of Exo-d-MAPPS in the alleviation of chronic lung inflammation. All of the 30 Exo-d-MAPPS-treated patients showed a marked improvement in pulmonary status, as evidenced by an increase in percentage change relative to the initial value of FEV1 (%ΔFEV1), significantly higher PEF, decreased CCQ total score, and increased 6-minute walking distance (6MWD) (Figures 6(b)–6(e)). Additionally, quality of life was significantly improved after Exo-d-MAPPS treatment and all Exo-d-MAPPS-treated patients managed to perform daily activities without hindrance. Clinical findings were confirmed by CT. Inflammation-induced destruction of alveoli and air trapping caused hyperinflation of the lungs with flattening of the diaphragm in COPD patients (Figure 6(f), red arrows). Exo-d-MAPPS significantly alleviated emphysematous changes in the lungs of COPD patients. Lungs were less hyperexpanded, diaphragms were less flattened, and centrilobular and paraseptal emphysema were significantly reduced one month after Exo-d-MAPPS administration (Figure 6(g), green arrows), indicating the beneficial effects of Exo-d-MAPPS in the attenuation of emphysema in COPD patients. Importantly, Exo-d-MAPPS was well tolerated. None of the 30 Exo-d-MAPPS-treated COPD patients reported any side effects related to Exo-d-MAPPS administration.

## 4. Discussion

MSCs are considered new therapeutic agents in the cell-based therapy of chronic inflammatory lung disorders due to their capacity to suppress detrimental immune response in the lungs and due to their ability to differentiate into alveolar cells, lung epithelial cells, and vascular and endothelial cells [39]. Nevertheless, results obtained in several animal models suggested that transplanted MSCs, in response to the growth factors produced in the local microenvironment, may also differentiate into undesired tissues, mainly bone and cartilage [40, 41]. Although MSCs have a low expression of major histocompatibility class (MHC) molecules, several lines of evidence indicated that transplantation of allogeneic MSCs can induce allogeneic immune responses in MHC-mismatched recipients [42–45]. Therefore, the safety issue related to MSC-based therapy is still a matter of debate [46].

Since most of the MSC-mediated beneficent effects in the attenuation of inflammatory lung diseases were a consequence of their paracrine action [36] and in order to avoid safety concerns related to unwanted differentiation of transplanted MSCs or their allogeneic rejection [40–45], we designed Exo-d-MAPPS, a PL-MSC-derived soluble product, which contains MSC-derived Exos with immunomodulatory factors involved in lung repair and regeneration (sTNFRI, sTNFRII, IL-1Ra, and sRAGE).

By using clinical data and a complementary animal model, herewith, we demonstrated the therapeutic potential of Exo-d-MAPPS in the alleviation of chronic airway inflammation. A marked improvement in pulmonary function and significantly attenuated airway inflammation were observed in COPD patients one month after Exo-d-MAPPS treatment. Similarly, a preserved alveolar structure and a diminished number of lung-infiltrated leucocytes were noticed in the lungs of CS+Exo-d-MAPPS-treated mice indicating that Exo-d-MAPPS efficiently alleviated inflammation-related pathological changes in the CS-injured lungs.

The beneficial effects of Exo-d-MAPPS in the alleviation of CS-induced COPD relied on the suppression of inflammatory immune cells in the lungs, and therefore, Exo-d-MAPPS was administered as an inhalation solution in COPD patients. Mice appear to lack respiratory bronchioles, partially alveolarized conducting airways, which are the sites of CS-induced emphysema [47]. Despite the species-specific difference in airway anatomy between mice and humans, mice have been the most usually used animal models for the investigation of the molecular and cellular mechanisms responsible for the therapeutic effects of newly developed drugs in COPD [48]. Among all routes for the daily application of the drugs, intraperitoneal administration is the most commonly used in mice since it is simple to perform and it results in a fast absorption of the drug into the vasculature [49]. On the contrary, during inhalational drug administration, mice should be conditioned to restraint devices and nose masks [49]. Stress, associated with physical restraint, has been shown to cause a negative impact on the cardiovascular and immune systems of mice and, accordingly, may alter the therapeutic effects of immunosuppressive agents, including Exo-d-MAPPS [50]. For these reasons, Exo-d-MAPPS was administered intraperitoneally in mice and by inhalation in COPD patients.

Alveolar epithelial cells exposed to CS produce TNF-*α* and IL-1*β* which bind to their receptors on lung endothelial cells (ECs) and induce increased expression of selectins and integrin ligands enabling massive accumulation of circulating leucocytes in the inflamed lungs [51]. Accordingly, TNF-*α* and IL-1*β* are considered inflammatory cytokines with the most important pathogenic role in the initial phase of lung injury and inflammation [51, 52]. Soluble TNF receptors (sTNFRI and sTNFRII) suppress TNF-*α*-driven chronic airway inflammation, and their serum and sputum levels positively correlate with lung function in COPD patients; therefore, sTNFRI and sTNFRII are considered important anti-inflammatory mediators responsible for lung repair and regeneration [53]. Similarly, MSC-derived IL-1Ra, a naturally occurring cytokine, acts as a competitive inhibitor of IL-1*β* and attenuates lung injury by preventing IL-1*β*-dependent accumulation of inflammatory cells in injured lungs [54]. IL-1Ra binds to IL-1R on ECs and prevents proinflammatory events initiated by an IL-1*β* : IL-1R interaction, including enhanced influx of neutrophils, macrophages, and lymphocytes in inflamed lungs [54]. In line with these findings, we assume that sTNFRI, sTNFRII, and IL-1Ra, which were present in high concentration in the Exo-d-MAPPS sample, were responsible for downregulated serum levels of TNF-*α* and IL-1*β* and for the significantly reduced presence of inflammatory TNF-*α*-producing macrophages and TNF-*α*- and IL-1*β*-producing neutrophils in the lungs of CS+Exo-d-MAPPS-treated animals.

NK and NKT cells promote lung injury and inflammation either directly, by inducing apoptosis of alveolar epithelial cells, or indirectly, through the secretion of inflammatory cytokines (IL-17A and IFN-*γ*), which regulate the accumulation and activation of inflammatory macrophages in the CS-injured lungs [55]. Accordingly, the attenuated injury of alveolar epithelial cells, noticed in the lungs of Exo-d-MAPPS-treated CS-exposed mice, was accompanied by the remarkably reduced presence of IL-17A- and IFN-*γ*-producing lung-infiltrated NK and NKT cells and significantly lower number of inflammatory M1 macrophages.

Exposure of alveolar epithelial cells to inflammatory cytokines results in the generation of oxidative stress which leads to the accelerated formation and accumulation of advanced glycation end products (AGEs). AGEs bind to their receptors (RAGEs) and cause lung injury through the formation of crosslinks within protein molecules [56]. Accordingly, the increased expression of AGEs and RAGEs was observed in the lungs of COPD patients, suggesting their important pathogenic role in the development and progression of chronic airway inflammation [57]. On the contrary, sRAGE acts as a decoy receptor which removes circulating AGEs, prevents their ligation to membrane bound RAGEs, and protects lung tissue from injury [58, 59]. Additionally, sRAGE suppresses AGE : RAGE-dependent production of inflammatory cytokines in mononuclear cells and promotes the generation of alternatively activated macrophages and regulatory DCs [60]. These anti-inflammatory cells have a reduced expression of MHC class II proteins and costimulatory molecules, induce the anergy of effector T cells, and promote the generation and expansion of immunosuppressive Tregs [61, 62]. In line with these findings, we assume that sRAGE, which was found in high concentration in Exo-d-MAPPS samples, was responsible for the reduced number of CD80 and I-A-expressing macrophages and DCs and for the increased presence of immunosuppressive, IL-10-producing alternatively activated M2 macrophages, regulatory DCs, and Tregs in the lungs of CS+Exo-d-MAPPS-treated mice.

IL-10-producing cells created an immunosuppressive microenvironment in the lungs of CS+Exo-d-MAPPS-treated animals and suppressed detrimental TNF-*α*, IFN-*γ*, and IL-17-driven T cell immune response. It is well known that TNF-*α*-, IFN-*γ*-, and IL-17-producing CD8+CTLs and CD4+ T helper cells have an important pathogenic role in the development and progression of COPD [61]. Lung-infiltrated CD8+CTLs induce the cell death of alveolar epithelial cells either directly, through the secretion of cytotoxic molecules (TNF-*α*, perforins, and granzymes), or indirectly, through the production of IFN-*γ* or IL-17 which promotes tissue destruction by promoting the secretion of matrix metalloproteinases (MMPs), TNF-*α*, and IL-1*β* in macrophages and neutrophils [63, 64]. In a similar manner, IFN-*γ*- or IL-17-producing CD4+Th1 and Th17 cells orchestrate immune response in inflamed lungs by amplifying the activation of lung-infiltrated inflammatory macrophages and neutrophils [61, 63]. Additionally, CD4+ T cells promote long-term survival of CD8+CTLs and are essential for the full development of CTL-mediated cytotoxicity in inflamed lungs [61, 63]. Accordingly, we believe that the Exo-d-MAPPS-induced expansion of lung-infiltrated IL-10-producing macrophages, DCs, and Tregs and elevated serum levels of IL-10 were responsible for attenuated T cell-driven airway inflammation and reduced injury of alveolar epithelial cells which were observed in CS+Exo-d-MAPPS-treated animals,

## 5. Conclusions

In summary, we propose that the main mechanism of action responsible for Exo-d-MAPPS-based alleviation of COPD relied on anti-inflammatory effects of soluble mediators (sTNFRI and II, IL-1Ra, and sRAGE) which inhibited the influx of inflammatory leukocytes and promoted the expansion of immunosuppressive cells in the lungs. Alteration in the cellular makeup of the lungs resulted in the creation of an anti-inflammatory microenvironment which enabled enhanced tissue repair and regeneration and improved pulmonary function of CS-exposed animals and COPD patients. Therefore, we believe that Exo-d-MAPPS could be considered a potentially new therapeutic agent in the treatment of chronic inflammatory lung diseases whose efficacy should be further explored in large clinical trials.

## Data Availability

The data used to support the findings of this study are included within the article.
